# Outflow tract septation and the aortic arch system in reptiles: lessons for understanding the mammalian heart

**DOI:** 10.1186/s13227-017-0072-z

**Published:** 2017-05-10

**Authors:** Robert E. Poelmann, Adriana C. Gittenberger-de Groot, Marcel W. M. Biermans, Anne I. Dolfing, Armand Jagessar, Sam van Hattum, Amanda Hoogenboom, Lambertus J. Wisse, Rebecca Vicente-Steijn, Merijn A. G. de Bakker, Freek J. Vonk, Tatsuya Hirasawa, Shigeru Kuratani, Michael K. Richardson

**Affiliations:** 1 Department of Cardiology, Leiden University Medical Center, Albinusdreef 2, Leiden, The Netherlands; 2 Animal Sciences and Health, Sylvius Laboratories, University of Leiden, Sylviusweg 72, Leiden, The Netherlands; 3 Department of Anatomy and Embryology, Leiden University Medical Center, Einthovenweg 20, Leiden, The Netherlands; 4 Naturalis Biodiversity Center, Darwinweg 2, Leiden, The Netherlands; 5 Laboratory for Evolutionary Morphology, RIKEN, 2-2-3 Minatojima-minami, Chuo-ku, Kobe, Hyogo 650-0047 Japan

**Keywords:** Cardiac development, Outflow tract cushions, Aorto-pulmonary septation, Flow divider, Neural crest, Second heart field, Reptiles, Crocodile, Bird

## Abstract

**Background:**

Cardiac outflow tract patterning and cell contribution are studied using an evo-devo approach to reveal insight into the development of aorto-pulmonary septation.

**Results:**

We studied embryonic stages of reptile hearts (lizard, turtle and crocodile) and compared these to avian and mammalian development. Immunohistochemistry allowed us to indicate where the essential cell components in the outflow tract and aortic sac were deployed, more specifically endocardial, neural crest and second heart field cells. The neural crest-derived aorto-pulmonary septum separates the pulmonary trunk from both aortae in reptiles, presenting with a left visceral and a right systemic aorta arising from the unseptated ventricle. Second heart field-derived cells function as flow dividers between both aortae and between the two pulmonary arteries. In birds, the left visceral aorta disappears early in development, while the right systemic aorta persists. This leads to a fusion of the aorto-pulmonary septum and the aortic flow divider (second heart field population) forming an avian aorto-pulmonary septal complex. In mammals, there is also a second heart field-derived aortic flow divider, albeit at a more distal site, while the aorto-pulmonary septum separates the aortic trunk from the pulmonary trunk. As in birds there is fusion with second heart field-derived cells albeit from the pulmonary flow divider as the right 6th pharyngeal arch artery disappears, resulting in a mammalian aorto-pulmonary septal complex. In crocodiles, birds and mammals, the main septal and parietal endocardial cushions receive neural crest cells that are functional in fusion and myocardialization of the outflow tract septum. Longer-lasting septation in crocodiles demonstrates a heterochrony in development. In other reptiles with no indication of incursion of neural crest cells, there is either no myocardialized outflow tract septum (lizard) or it is vestigial (turtle). Crocodiles are unique in bearing a central shunt, the foramen of Panizza, between the roots of both aortae. Finally, the soft-shell turtle investigated here exhibits a spongy histology of the developing carotid arteries supposedly related to regulation of blood flow during pharyngeal excretion in this species.

**Conclusions:**

This is the first time that is shown that an interplay of second heart field-derived flow dividers with a neural crest-derived cell population is a variable but common, denominator across all species studied for vascular patterning and outflow tract septation. The observed differences in normal development of reptiles may have impact on the understanding of development of human congenital outflow tract malformations.

## Background

In mammals, the normal formation of the aorto-pulmonary septal complex has been analysed mostly in the setting of the description of mutant mice or manipulated avian embryos, resulting in outflow tract malformations including malalignment of the outflow tract (OFT) septum, asymmetric arterial development (Tetralogy of Fallot), transposed great arteries (TGA) and absent separation of the great arteries, resulting in a persistent truncus arteriosus (common arterial trunk) [1–3]. It is evident that remodelling of the myocardial OFT at the junction with the vascular aortic sac is complex and employs different cell populations (myocardium, endocardial cushions, neural crest and mesenchymal second heart field) to various degrees [4–6]. Here, we concentrate mostly on three levels of the developing heart, the proximal outflow tract (also called the conus arteriosus), the distal outflow tract (truncus arteriosus) and the pharyngeal arch arteries emerging from the aortic sac. The remodelling of the branchial or pharyngeal arch system is one of the hallmarks of amniote development. The pharyngeal arches encompass key structures including neuronal, supportive, muscular and vascular elements together making up parts of the face, neck and upper thoracic region [7]. The pharyngeal arch arteries (PAAs) in reptiles and birds (sauropsids) and in mammals develop in a craniocaudal sequence as shunts between the aortic sac and the paired dorsal aortae. The mode of PAA remodelling is essential for understanding OFT separation connecting the aortic sac to the non-septated ventricular (most reptiles) or biventricular heart (crocodiles, mammals and birds). The latter results invariably in one single pulmonary trunk, dividing into a right and left pulmonary artery (PAA6), connected to the right ventricle, or the right-sided cavum pulmonale of the common ventricle. In the species studied, this is combined with two aortae (left and right PAA4) in the lizard, turtle, crocodile and early embryonic bird. The left visceral aorta arises from the cavum venosum of the common ventricle, whereas the right systemic aorta emerges from either the left ventricle, or the cavum venosum receiving blood from the cavum arteriosum. In birds, the left PAA4 disappears early in development leaving a right PAA4 which forms a right-sided aortic arch in the mature animal. In crocodiles, the right PAA4 arises from the left ventricle; this becomes the systemic aorta, supplying, for example, the cranial and brachial regions and the body wall with oxygen-rich blood. However, the left PAA4, supplying the viscera, arises from the right ventricle together with the pulmonary trunk. Therefore, the left PAA4 has also been called the visceral aorta [8]. In mammals, the aortic trunk arises from the left ventricle and the left PAA4 will form the left-sided aortic arch, while the right PAA4 forms the basis of the right subclavian artery.

Remodelling of the OFT and the aortic sac with the emerging PAAs requires the involvement of several cell populations. First of all, extracardiac cells from the postotic rhombencephalic neural crest contribute to this area [9, 10]. Furthermore, at this level the vessels are embedded in the second heart field [11–13], which is part of the splanchnic mesoderm, encompassing also the coelomic lining of the pericardial cavity that gives origin to the arterial epicardial cells [14, 15]. The synchronized development of these cell populations results in the formation of OFT myocardium and endocardial cushions, in conjunction with the aorto-pulmonary septum [4–6] and the wall of the arterial trunks [2, 10, 16–18] in a narrow time window.

Ventricular septation is a complex process, involving not only the myocardial wall but also the overlying epicardium. Recently, we distinguished the ventral part of the interventricular septum as the folding septum [19]. As it is located adjacent to the outflow tract, the relation with the proximal conal endocardial cushions, more specifically the septal cushion, is of interest. Not only does the separation at intracardiac and arterial level show variations, but the remodelling in the distal segments of the PAAs is also variable between species. In all taxa investigated, the connection of PAA3 with the paired dorsal aortae (carotid ducts) disappears. In reptiles including birds, the left and right PAA6 (ductus arteriosus), connecting to the dorsal aortae, persist until hatching. In many reptiles, they persist even after hatching as in *Sphenodon*, Testudines, several species of lizards and snakes, and maybe even in some crocodiles; for detailed comparative descriptions, see [20–23]. In mammals, the right PAA6 disappears early in development, while the left PAA6 persists until birth as the ductus arteriosus of Botalli [24, 25]. Although complete anatomical septation at the level of the left and right ventricle takes place in crocodiles, both anatomical and functional separation of pulmonary and systemic blood flows remain incomplete, as a central shunt between the roots of both aortas provides a direct communication between left and right ventricular outflows. The development of this foramen of Panizza (as first described by Bartolomeo Panizza in 1833, see Ref. [8]) adds to the complexity of the crocodilian circulation.

This complex diversification resulting from the evolutionary remodelling of a symmetric ancestral PAA system including the (septating) heart has been used to provide characteristics in phylogeny reconstruction [7, 20, 23, 26, 27]. The position of the Testudines, for example, has been contentious, because embryological, morphological and molecular data can yield conflicting phylogenies. Thus, it has been variously suggested that turtles are a basal reptilian clade [28], a sister group to lepidosaurs [29] or sister group to the archosaurs (together forming the Archelosauria) [30–34].

Basic to the circulatory system is the beating heart providing the propelling force for the arterial blood flow, and the resulting haemodynamic forces are in themselves an important modifier of development [35–38], allowing shear stress responsive genes such as endothelin1, KLF2 and NOS3 to enter the stage [39]. Modulation of these and other genes in mouse models often results in cardiovascular malformations.

The aim of this study is to examine, in a comparative evolutionary developmental biology context: (1) the respective roles of various cell populations (second heart field, endocardial and neural crest cells) in the morphogenesis of both the interaortic flow divider and the aorto-pulmonary septum between the aortic and the pulmonary trunks so as to produce three arterial trunks from the aortic sac: the left (visceral) and right (systemic) aorta as well as the pulmonary trunk; (2) the cellular mechanisms underlying the septation of the intracardiac outflow tract in reptiles, which is minimal in lizards and turtles and becomes myocardialized in crocodiles and birds.

We use an integrative ‘evo-devo’ approach encompassing comparative morphology, developmental biology and protein expression patterns in embryonic cell populations. Specifically, we apply (immuno-)histochemistry to serially sectioned embryos and implement Amira-based 3-D reconstructions to reveal the spatiotemporal remodelling of the outflow tract and PAAs. We have applied this approach by sampling the following taxa: agamid lizards (bearded dragon, *Pogona vitticeps*), Testudines (the soft-shell turtle *Pelodiscus sinensis*), crocodiles (Nile crocodile, *Crocodylus niloticus*), and birds (chicken, *Gallus gallus*). We analyse our findings also in the context of what is described in the literature about the corresponding processes in mammals. In mammals [2, 18] and birds [9, 10, 12], the dual contribution of both second heart field and neural crest to the aortic root and the aorto-pulmonary septal complex has been described. We will demonstrate that in birds the position of the aortic flow divider and in mammals the pulmonary flow divider are essential for understanding the formation of the aorto-pulmonary septal complex.

## Materials and methods

*Pelodiscus sinensis* eggs were purchased from a Japanese local farm and incubated at 30 °C, and the embryos were fixed at different developmental stages at the Evolutionary Morphology Laboratory, RIKEN (Kobe, Japan). The crocodile embryos were obtained from La Ferme aux Crocodiles (Pierrelatte, France). Bearded dragons were obtained through local breeders, and specific pathogen-free chicken eggs from a commercial source.

*Normal stages* were studied of chicken (*G. gallus,* HH17–37, *N* = 24), bearded dragon (*P. vitticeps, HH22*–*36, N* = 18), Chinese soft-shell turtle (*P. sinensis,* HH19–32, *N* = 9), Nile crocodile (*Crocodilus niloticus* HH19–40, *N* = 14). Although specific developmental descriptions of external characteristics of various reptilian species are available [40–44], we staged all these species according to the Hamburger–Hamilton (HH) stages for the chicken [45], for ease of comparison (Table 1).

**Table 1:** Comparison of the stages of the various species studied

Chicken	Anolis	Pelodiscus	Alligator
Hamburger	Sanger	Tokita	Ferguson
19/20		12	10
22	6		12
24	7		13
25/26	8	15	14
27		16	16
28–30	9/10	18	17
31/32	11	19	19
33/35	12		20/21

We used the stages provided by Sanger et al. [40] (*Anolis* comparable to *Pogona*), Tokita and Kuratani [43] (*Pelodiscus*) and Ferguson [42] (*Alligator* for *Crocodylus*). Our experience reveals this to be practical for use in comparing cardiovascular development between reptiles, notwithstanding differences in developmental stages between species due to heterochrony in several organ systems [46], which becomes particularly evident after approximately HH stage 32.

### Fixation and histology

Embryos were fixed for 24–48 h in buffered 4% paraformaldehyde at 4 °C. They were stored in methanol 100% at −20 °C until further use. Chicken embryos were stored in 70% ethanol at 4 °C. Subsequently, the embryos were transferred to 100% ethanol and embedded in paraffin with Histo-Clear II (National Diagnostics, Atlanta, Georgia, USA) as the intermediate reagent. Thoracic blocks of tissue, containing the heart and pharyngeal arch arteries, were serially sectioned (5 µm) and mounted on objective slides allowing for five different sequential stainings of sister sections. As a standard, one set of sister sections was stained with hematoxylin–eosin. In addition, selected specimen were stained using the Movat or Sirius red procedure to demonstrate components such as elastin, collagen, glycosaminoglycans (GAGs) and (smooth) muscle (immunohistochemistry was also used as described below).

### Staining procedures

Paraffin sections were histologically stained according to slightly modified procedures as described in Bancroft and Gamble (6th ed. 2008). This holds for the haematoxylin–eosin staining, the Russell modification of the Movat Pentachrome staining and the Sirius Red staining.

### Immune incubations

We chose to use immunohistochemical staining protocols as these can be applied to the fixed and variously stored material of the species under study. Adjacent sister sets of sections were stained with a selection of antibodies for MLC2a (myosin light chain for myocardium) or CTNI (cardiac troponin I for myocardium); homeobox protein NKX2–5 (myocardial cells and SHF cardiac precursors), HNK1 for early migrating neural crest cells, the transcription factor TFAP2α (activator of protein, for migrating neural crest cells) [4], Isl1 for second heart field cells, WT1 (Wilms tumour-like 1 for embryonic mesothelium (epicardium, pericardium) including associated mesenchymal cells [47, 48].

Paraffin sections for immune incubations were dewaxed in Histo-Clear, rehydrated via a decreasing percentage of ethanol and microwaved for 12 min in 0.01 M citric buffer pH 6.0 for antigen retrieval. Endogenous peroxidase was inhibited using 0.3% H_2_O_2_ in phosphate-buffered saline (PBS) for 20 min and the sections rinsed in PBS.

For staining sections, the first antibodies were diluted in BSA/PBS and incubated overnight at room temperature (Isl1, 1/100;CTNI, 1/800; NKX2–5 1/2000; TFAP2α 1/500; WT1 1/3000; MLC2a 1/6000; HNK1, 1/10). The WT1 staining was performed on freshly sectioned material. The antibodies were raised in rabbit, except for NKX2.5 (in goat) and Isl1 and HNK1 (in mouse).

The second antibody depended on the species in which the first antibody was obtained, and was applied as follows: Isl1: 60-min horse anti-mouse 1/200 in horse serum; CTNI: 60-min goat anti-rabbit-biotin, 1/200 in goat serum; NKX2.5: 60-min horse anti-goat-biotin 1/66 in horse serum; TFAP2α: 45-min goat anti-rabbit biotin 1/200 in goat serum; WT1: 60-min goat anti-rabbit biotin 1/200 in goat serum; MLC2a: 60-min goat anti-rabbit biotin 1/200 in goat serum. HNK1: 120-min rabbit-anti-mouse 1/250 in bovine serum.

The procedures were finalized by ABC reagents (avidin-biotinylated complex) according to manufacturers’ protocol: 45 min. Visualization took place by DAB/H_2_O_2_ (10 min) followed by rinsing in demineralized water. Sections were briefly counterstained with haematoxylin, dehydrated and coverslipped with Entellan.

Primary antibodies were obtained from Vector labs (Isl1, second antibodies, ABC reagents), Santa Cruz (CTNI, NKX2–5, WT1), Gene Tex (TFAP2α), and Hybridoma Bank (HNK1).

### Image processing

Images of high quality and resolution were created using the Philips Ultra Fast Scanner 1.6 (Dept. Pathology, Leiden University Medical Centre (LUMC), Leiden). Images were imported into the database Philips Image Management System (IMS), which has the benefits of working digitally and allow to view multiple images simultaneously. The selected material for 3D reconstruction all met the following criteria: clearly visible (immuno)staining, no important structures for heart development missing, and sections were positioned in the correct order to follow-up for Amira. Construction of the 3-D models was done in Amira version 5.3.3 (FEI Visualization Sciences Group, Bordeaux). First, the images were optimized by cropping and enhancing the contrast. Voxel size was determined, and images were loaded into Amira. After that, the various structures were selected and reconstructed three-dimensionally. Segmentation of the structures studied was based on a combination of histology and immune staining patterns. Finally, reconstructions were converted into 3-D pdfs allowing study of the whole reconstruction database or of a subset of elements, as, for instance, only the pharyngeal arch arteries.

**Glossary: Definition of terms used for vessel segments**

PAA1–6: Pharyngeal arch arteries 1–6
Aortic sac: Common vessel connecting the myocardial OFT to the respective arterial trunks
Carotid trunk: Combined stem of the left and right PAA3, joining the right PAA4
Carotid duct: (Disappearing) segment of the dorsal aorta between PAA3 and PAA4
Aortic trunk: Combined stem of the left and right PAA4
Pulmonary trunk: Combined stem of the left and right PAA6, emerging from the right ventricle or cavum pulmonale
Systemic aorta (sAo): Right-sided PAA4, emerging from the left ventricle or the cavum venosum-cavum arteriosum combination
Visceral aorta (vAo): Left-sided PAA4, emerging from the right ventricle or cavum venosum; this artery disappears early in birds
Ductus arteriosus: PAA6 connecting the pulmonary trunk to the respective dorsal aortae, the right-sided PAA6 disappears early in mammals.

## Results

### Notes on the OFT cushions

In the myocardial conus arteriosus (proximal outflow tract), we encounter 1 or 2 cushions, depending on the species: the septal cushion flanking the (interventricular) folding septum, which is not always accompanied by the parietal cushion as the latter is absent in squamates (lizards and snakes). In the myocardial truncus arteriosus (distal OFT), usually 4 cushions are encountered, the large septal cushion along the continuation of the folding septum (which is part of the interventricular septum) and continuous with the conal septal cushion; furthermore, the parietal cushion and 2 intercalated cushions, the left and right one. The fused septal and parietal cushions may show myocardialization, particularly evident in archosaurs (crocodiles and birds), being absent in squamates and incomplete in *Pelodiscus* (turtle). A comparison of the important features relating to arterial development, OFT septation and cell population participation in the different species is provided in Table 2.

**Table 2:** Summary of principal differences

	Lizard	Turtle	Crocodile	Bird	Mammal
Short left visceral aorta	✓	✓	✓	×^a^	×
Short right systemic aorta	✓	✓	✓	✓	×
Long aortic trunk, bifurcating	×	×	×	×	✓
Unseptated outflow tract	✓	✓	×	×	×
Completely septated ventricles	×	×	✓^a^	✓	✓
Parietal OFT cushion in conus	×	×^b^	✓	✓	✓
SHF in PAA4 and PAA6 flow divider	✓	✓	✓	×^b^	✓
AP septal complex	×	×	×	✓^c^	✓^d^
NCC in muscular AP septum	×	×	✓^e^	✓	✓
NCC (TFAP2α) in PAA3	×	✓	✓^f^	nd	nd
Foramen of Panizza (FOP)	×	×	✓^g^	×	×

✓, present; ×, absent

*nd* not determined, *AP* NC-derived aorto-pulmonary septum

^a^Left PAA4 disappears early in development

^b^Appears later in development

^c^PAA4 SHF-derived flow divider

^d^PAA6 SHF-derived flow divider

^e^The septum is mostly muscularized

^f^TFAP2α less distinct in crocodile than in turtle

^g^Specific character of species

#### Chicken (*G. gallus*)

**Fig. 1:**
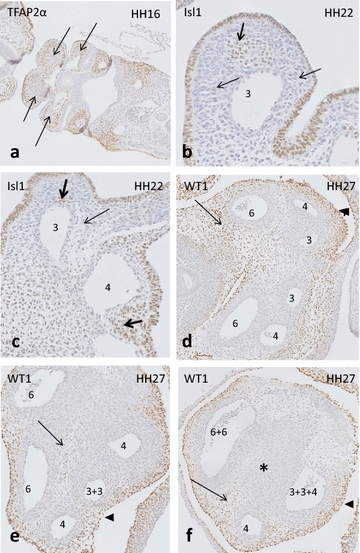
Early development of the pharyngeal arches in chicken. **a** HH16 TFAP2α (*brown staining*) present in NCC, the negative core of pharyngeal arches is indicated (*arrows*). **b**, **c** HH22, Isl1 positive core of the pharyngeal arch (*bold arrow*); the NCC area of the arch and the arterial wall are negative (*thin arrows*). **d**–**f** HH27 from distal (**d**) to proximal (**f**), WT1 in the coelomic lining (*arrowhead*) and the mesenchyme are positive (*arrowheads*). Note NCC-derived arterial walls and condensed mesenchyme (* in **f**) are WT1 negative. Numbers *3*, *4* and *6* indicate the various PAAs

In **HH16** PAA1, 2 and 3 are embedded in their respective pharyngeal arches. HNK1 staining is positive in the early migrating neural crest (NCC) cells adjacent to the neural tube (not shown), while TFAP2α staining includes also a later population of NCC in the mesoderm of the pharyngeal arches. The NCC only reaches the heart after HH19. A negative core in the pharyngeal arches reveals a non-NCC population, which presumably consists of second heart field (SHF)-derived cells (Fig. 1a).

In later stages (**HH22** and onwards), the number of TFAP2α staining cells (NCC) in the mesenchyme of the pharyngeal arches diminishes dramatically, due to downregulation in the differentiating NCC, as specific cells in the neural tube remain positive, proving that the staining protocols are adequate. Therefore, in the chicken we used alternative markers to distinguish NCC from second heart field-derived cells. To that aim the second heart field ‘marker’ ISL1 was used, leaving NCC negative (Fig. 1b, c). Similarly, WT1 marking the coelomic epithelial lining and its mesenchymal cells allows visualization of non-NCC in the arterial pole (Fig. 1d–f).

**Fig. 2:**
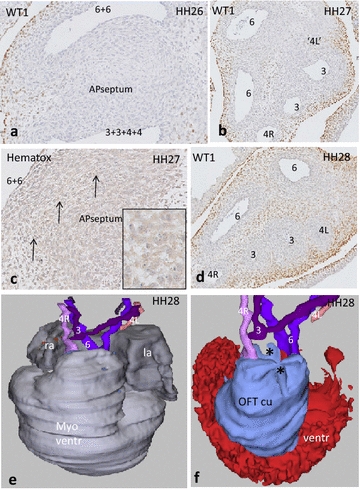
Development of the AP septum in chicken HH26–28. **a** The AP septum between Pu (6 + 6) and Ao channels (3 + 3 + 4 + 4). WT1 staining (*brown*) in the epicardium and mesenchymal second heart field cells. **b** slightly more distal, the Pu is separated into the left and right PAA6 and the Ao channel in the right PAA4 and separate PAA3. Note that the lumen of the left PAA4 has disappeared. **c** The area of apoptosis is continuous with the massive apoptosis in the AP septum (*arrows* and *inset*, see also Fig. 4e). **d** In HH28, the lumen of the *left* PAA4 (4L) is nearly occluded and the vascular smooth muscle cells (negative in this WT1 staining) are still visible. Note the elaborate WT1 staining (*brown*) between the vascular segments. **e** Amira reconstruction of HH28 seen from ventral showing the lumina of the arterial trunks. *la* left atrium, *ra* right atrium, myo ventr myocardium ventricle **f** Same embryo, the fused OFT cushions (*blue*) contain a groove for the condensed mesenchyme (*) running between PAA6 (6) and the stem of PAA4R (4R) and PAA3 (3). Note that the lumen of PAA4L (4L) is interrupted and does not reach the heart anymore. OFT cu fused outflow tract cushions, ventr lumen ventricle

In stage **HH26** and **27,** the dorsal aorta is still surrounded by Isl1 and WT1 (Fig. 2a) positive mesenchymal cells, but walls of the PAA are negative. PAA walls are also negative for TFAP2α (not shown). The lumen of the left PAA4 adjacent to the heart becomes diminished and even occluded (Fig. 2b), appearing again as an open stub distal to the OFT (compare with Fig. 2d). The wall of the regressing left PAA4 contains apoptotic cells in a zone that extends into the condensed mesenchyme of the aorto-pulmonary septum (Fig. 2c). Proximally in the OFT, two endocardial cushions (septal and parietal) are present; more distally these two cushions fuse, while the NC-derived condensed mesenchyme enters the fused cushion complex, where it comes to occupy a position exactly between the aortic and pulmonary channels (Fig. 2e, f). Although the main body of condensed mesenchyme is located inside the myocardial border, part of it remains in the mesenchyme of the arterial pole. The condensed mesenchyme particularly on the dorsal side is adjacent to WT1-expressing mesenchymal cells, which are spatially continuous with the overlying WT1-positive coelomic lining cells (Fig. 2d).

The carotid trunk (the common left and right PAA3) together with the right PAA4, but excluding the disappearing left PAA4, shares a common stem (aortic trunk) upon leaving the heart, and this aortic trunk is parallel to the pulmonary trunk (Fig. 2f). The separated left and right PAA6 are asymmetrically located where they leave the heart. In particular, the right PAA6 changes position to dorsal and right. It is important to stress that both PAA6 persist until hatching, whereas in mammals only the left PAA6 persists until birth.

**Fig. 3:**
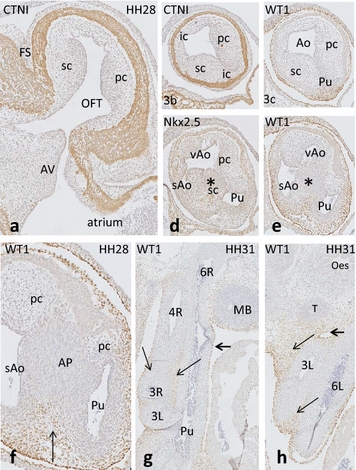
The OFT cushions in chicken HH28. The endocardial outflow tract (OFT) cushions from proximal to distal in adjacent sister sections. The septal (sc) and parietal cushion (pc) are indicated. **a** Both cushions appose each other in the proximal OFT. **b**, **c** They come close to each other, the Ao and Pu channels are indicated. **d** The sc and pc have fused showing here the NCC-derived condensed mesenchyme (c*). **e** The Ao channel is divided into the left visceral (vAo) and right systemic aortic (sAo) channels. **f** The pc shows 2 segments on both sides of the condensed mesenchyme, WT1-positive cells dorsally to the AP septum (*arrow*). **g**, **h** Chicken HH31. The left PAA4 has completely regressed from the OFT. Only both PAA3, PAA4R and PAA6 are present. Pu indicates the stem of both PAA6, of which PAA6R is present in **g** and PAA6L in **h**. WT1 staining reveals the mesenchyme cells surrounding the negative tunica media of the arteries. *Thin arrows* indicate the WT1-positive mesenchyme between the arteries. The different positions of the left and right PAA6 leave an asymmetric pericardial recesses dorsal to the arterial pole (*bold arrow*). *Ao* aortic trunk, *AP* aorto-pulmonary septum, *AV* atrioventricular cushions, *FS* folding septum, *ic* intercalated cushion, *MB* main bronchi, *Oes* oesophagus, *pc* parietal cushion, *Pu* pulmonary trunk, *sAo* right systemic aorta, *sc* septal cushion, *T* trachea, *vAo* left visceral aorta

In the proximal OFT at **HH28**, the parietal and septal endocardial cushions are present (Fig. 3a). In the distal OFT (Fig. 3b–e), the left and right intercalated cushions complete the set of four distal OFT cushions (Fig. 3b). SHF-derived mesenchymal cells are located in the flow dividers between both PAA6, and between the right PAA4 and the remainder of the left one. Furthermore, SHF cells are found dorsal to, but not inside, the condensed mesenchyme between the pulmonary trunk (and both pulmonary arteries) and both aortic stems (Fig. 3e). As a consequence, NCC (Fig. 3d, e) participates only in the aorto-pulmonary septum between the aortic and pulmonary trunks, whereas SHF mesenchymal cells are located in the periphery of the aorto-pulmonary septum (Fig. 3f).

In **HH31–33,** the aorto-pulmonary septum still carries apoptotic cells between the left and right ventricular OFTs. OFT septation is now completed. Thick semilunar valve leaflets are present. The pulmonary and aortic trunks are separated, and mesenchymal WT1-positive cells are still present between the aortic and pulmonary arteries (Fig. 3g, h). A coronary artery ostium is present in the aortic root at the sinus of Valsalva (not shown). Remodelling of the PAAs is complete.

#### Bearded dragon (*P. vitticeps*)

Development of the OFT and PAAs in the bearded dragon shows a number of differences from that described above for the chicken. Most obvious are the absence of a cardiac interventricular septum, and the persistence of both the left visceral and right systemic aorta in addition to the pulmonary trunk (for a summary of the major features, see Table 2).

**Fig. 4:**
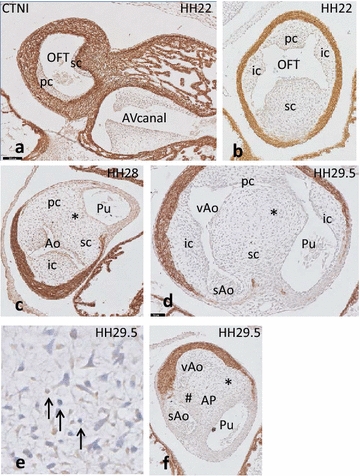
OFT development in the lizard *Pogona* HH22–29.5. **a** In the proximal OFT a thin cellularized septal cushion, flanking the folding septum and the equally indistinct parietal cushion are present. *Bar* = 100 µm. **b** The centrally located septal (sc) cushion is flanked by the parietal (pc) and intercalated (ic) cushions. Note that the condensed mesenchyme in the septal cushion is inconspicuous. **c** In HH28, the septal and parietal cushion have fused (*), separating the aortic and pulmonary channels. **d** At HH29.5, the aortic channel is not divided, yet, but a left visceral aorta (vAo) and a right systemic aorta (sAo) can be discerned. *Bar* = 50 µm. **e** Higher magnification shows some apoptotic cells in the septal cushion. **f** The aorto-pulmonary septum (*) separates the two aortic from the pulmonary channels, while a second separation (#) is present between both aortae. No condensed mesenchyme is visible in the AP septum. Abbreviations as in Fig. 3

In **HH22/23,** the proximal OFT contains relatively indistinct endocardial cushions, i.e. a cellularized septal cushion flanking the folding septum together with the opposing parietal cushion (Fig. 4a). In the distal OFT, four distinct cushions are present (Fig. 4b). Some apoptotic cells are present in the core of the parietal and the septal cushion. There is no fusion of cushions in this stage.

In **HH26,** three arterial stems emerge from the aortic sac; these are the pulmonary, the aortic and the carotid trunks. Shortly after leaving the aortic sac, these trunks divide symmetrically into their arteries comprising PAA 6, 4 and 3, respectively. They terminate in the left and right dorsal aortae.

In the next stage **(HH28),** both septal and parietal cushions, connected to each other by a thickened cellularized endocardium, are present in the proximal OFT. Distally, four cushions are present, although not exactly at the same level. The septal cushion contains an inconspicuous whorl-like core, considerably less massive than the condensed mesenchyme seen in a comparable stage and location in the chicken. Neural crest participation in this whorl could not be found by TFAP2α staining. Where the myocardial sleeve meets the arterial walls, the septal cushion fuses asymmetrically with the broadened parietal cushion, separating the pulmonary and aortic channels (Fig. 4c). Slightly more distally the septal cushion again fuses asymmetrically, this time with a part of the lateral aortic intercalated cushion, dividing the left visceral and right systemic aorta.

In the distal outflow tract of **HH29.5,** the large septal cushion (Fig. 4d) contains an indistinct body of condensed mesenchyme, which shows a few apoptotic cells (Fig. 4e); these are less abundant than in the same location in the chicken heart. The septal cushion has fused in two places: first with the parietal (Fig. 4d) and next with the aortic intercalated cushion (Fig. 4f). The pulmonary intercalated cushion is not involved in OFT separation. Distal to the myocardium the pulmonary trunk shifts to a more dorsal position and splits into both PAA6 of which the right one reaches a more cranial axial level. In the oldest stages studied **(HH34, 36**), the condensed mesenchyme in the fused cushions remains as indistinct as in the earlier stages.

#### Chinese soft-shell turtle (*P. sinensis*)

**Fig. 5:**
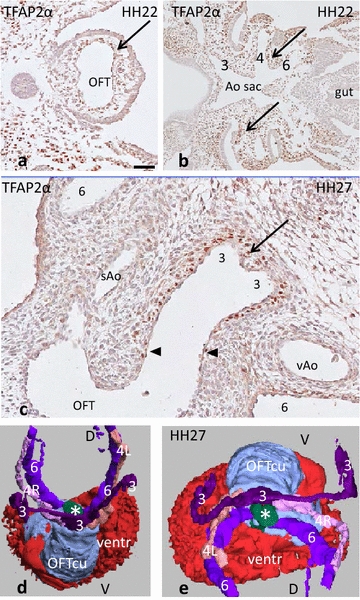
OFT development in the turtle *Pelodiscus* HH22–27. **a** TFAP2α+ cells (*brown*) in the pharyngeal mesoderm and the endocardiac jelly (*arrow*). Positive cells are only found over a short distance (<50 μm) into the heart. *Bar* = 100 µm. **b** In the same embryo, the periphery of the pharyngeal arch arteries also shows positive brown-stained cells (*arrows*). **c** HH27 TFAP2α in the wall of the truncus caroticus (PAA3, *long arrow*). There is no TFAP2a positivity in the OFT cushions beyond the border with PAA3 (*arrowheads*). TFAP2α-positive cells in the basis of the individual PAA3, but not in the sAo, the vAo or the PAA6. **d**, **e** Amira reconstruction of the ventricular lumen (*red*), OFT cushions (*blue*), NCC condensed mesenchyme (green*) and PAAs, indicated by colour and number (3, 4, 6). **e** Seen from cranial. OFT cu outflow tract cushions, ventr ventricular lumen, *D* dorsal, *V* ventral

In the youngest available embryo (**HH22),** the strongly looped OFT consists of a myocardial sleeve surrounding a uniformly distributed cardiac jelly. The latter contains mesenchymal cells, but is not organized into distinct cushions. In the distal OFT, a number of ventrally located subendocardial mesenchymal cells are TFAP2α positive, suggesting that they are NCC (Fig. 5a). Where the OFT enters the body wall, the aortic sac divides into three pairs of PAA embedded in the TFAP2α-positive mesenchyme of the pharyngeal arches; this demonstrates the clear presence of NCC in the branchial apparatus (Fig. 5b).

At **HH27,** the proximal OFT contains only a septal cushion and not a parietal one, although the subendocardium is cellularized. In the distal OFT, four cushions are present. Many cells in the tunica media of the carotid trunk including the branches of the left and right PAA3 stain heavily for TFAP2α (Fig. 5c), indicating the presence of NCC. The other PAAs do not present with positive cells. Likewise, the AP septum between pulmonary and aortic trunks is negative for TFAP2a, possibly downregulated in the NCC. A 3D reconstruction demonstrates the location of the condensed mesenchyme of the AP septum (green in Fig. 5d, e).

**Fig. 6:**
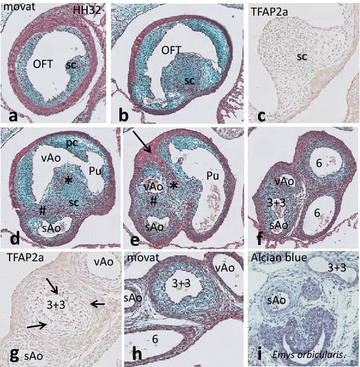
Development of the vascular segments in the turtle *Pelodiscus.* OFT cushions (from proximal to distal) and NCC contribution, movat and TFAP2α staining. **a** In the proximal OFT only the septal cushion is prominent between the cellularized cardiac jelly. **b** The septal cushion containing condensed mesenchyme becomes flanked by the other cushions. Here, a cell-rich whorl is present, pointed to the parietal cushion. **c** Magnification of an adjacent section, negatively stained for TFAP2α. **d** Another condensation (rich in SHF, #) separates both aortae. The myocardial wall on the dorsal side is retreating. **e** The three arterial trunks are separated. Note that a spur of myocardium (*arrow*) protrudes into the NCC area (*). **f** The carotid trunk (3 + 3) branches off the systemic aorta. The myocardial sleeve is not longer present. **g** Positive TFAP2α in the inner wall of the carotid trunk (3 + 3). **h** The inner media of the carotid trunk is rich in glycosaminoglycans compared to the adjacent arteries, giving it a spongy appearance. Abbreviations as in Fig. 3. **i** Cross section of the neck region of an embryo of *Emys orbicularis* showing the carotid trunk (3 + 3). The arterial wall is not spongy as in Pelodiscus (compare **h**). The *Emys* embryo belongs to material described earlier by our group [50]

**HH 30–32**: The septal cushion now extends the full length of the OFT (Fig. 6a–c). As in *Pogona*, it has fused twice, once with the parietal (Fig. 6d) and once with the aortic intercalated cushion (Fig. 6e). The site of fusion with the parietal cushion is located between both aortae and the pulmonary trunk; the site of fusion with the intercalated cushion is located between the visceral and systemic aorta (Fig. 6d, e). The enveloping myocardium protrudes inward to meet the NCC area between pulmonary trunk and aortae (Fig. 6e, arrow) but does not extend as far as the opposite dorsal side.

Although the septal cushion remains negative for TFAP2α (Fig. 6c), it shows condensed mesenchyme indicative of the presence of NCC probably differentiating into cartilage [49]. More downstream the pulmonary trunk divides into both PAA6 (Fig. 6f), while the left visceral aorta does not branch at all. The right systemic aorta gives off the carotid trunk, which is still positive for TFAP2α (Fig. 6g) and rich in extracellular matrix glycosaminoglycans giving it a spongy appearance (Fig. 6h). Both carotids branch into the left and right subclavian arteries (not shown) as is the case in all reptilian embryos of comparable developmental stage investigated here. Additionally, another turtle of which the cardiac development has been described by our group [50] was re-investigated for the embryonic architecture of the carotid artery. In *Emys orbicularis*, the carotid artery lacks the spongy appearance (Fig. 6i).

#### Crocodile (*C. niloticus*)

In **HH18,** the OFT cardiac jelly is sparsely cellularized; a septal and a less distinct parietal cushion is already present.

**Fig. 7:**
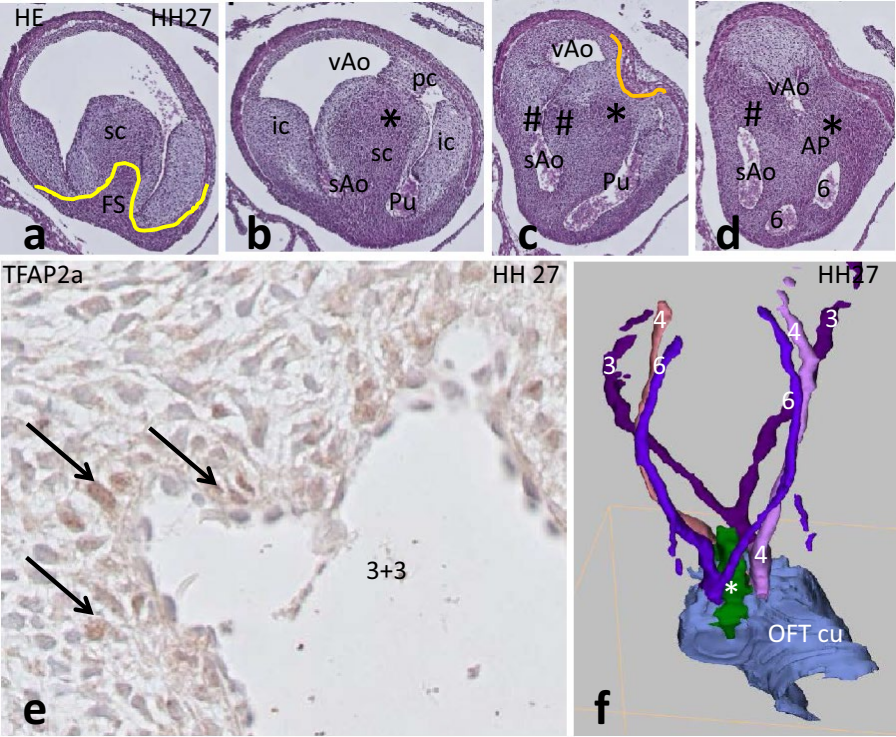
OFT development in *Crocodylus*. **a** In the proximal OFT the septal cushion flanking the folding septum (FS, *yellow curve*) is obvious. **b**–**d** Three consecutive sections in the distal OFT in which the OFT cushions are visible. The condensed mesenchyme of the aorto-pulmonary septum is indicated (*). In **c** at the arterial level the AP separates the pulmonary trunk from the aortic channels. The NCC (*) and SHF aorto-aortic flow divider (#) are indicated. At the ventral side, the myocardium protrudes inward (orange curve). **d** The arterial trunks are completely separated by the AP septum and the aortic flow divider (#). **e** TFAP2α-positive cells (NCC) in the wall of PAA3 (*arrows*), but not in the other PAAs (not shown). **f** 3D reconstruction of the fused OFT cushions and the PAAs (indicated by their *number*) including the AP septum (green*) between PAA3 + 4 and PAA6. Abbreviations as in Fig. 3

During the **HH 26**–**30** stages, the four endocardial cushions appear in the distal segment of the OFT (Fig. 7a, b). The septal cushion occupies a central position in the distal OFT (Fig. 7b, c). An elaborate whorl of condensed mesenchyme (Fig. 7a) fills the central and subendocardial aspect of the septal cushion and contains apoptotic cellular fragments. The twofold fused septal cushion separates the arterial trunk into three flow channels, providing for the systemic aortic trunk, the visceral aorta and the pulmonary trunk (Fig. 7c, d). The systemic trunk branches into the right PAA4 and the carotid trunk as in the other reptiles studied. Upon splitting into the left and right carotid arteries, TFAP2α-positive cells appear in the vessel wall (Fig. 7e), both in the tunica media and in the more peripheral adventitia, confirming the presence of NCC. Spinal ganglia and the vagus nerves also contain TFAP2α-positive cells. On both sides between PAA4 and PAA6, two vascular remnants were observed; these are probably vestiges of a PAA5, adjacent to pharyngeal pouch IV. The right PAA5 ends blindly in the mesenchyme, whereas the left one merges again with PAA6. A 3D reconstruction seen in dorsal aspect demonstrates the central position of the condensed mesenchyme of the AP septum between PAA 6 and both PAA4 (Fig. 7f).

**Fig. 8:**
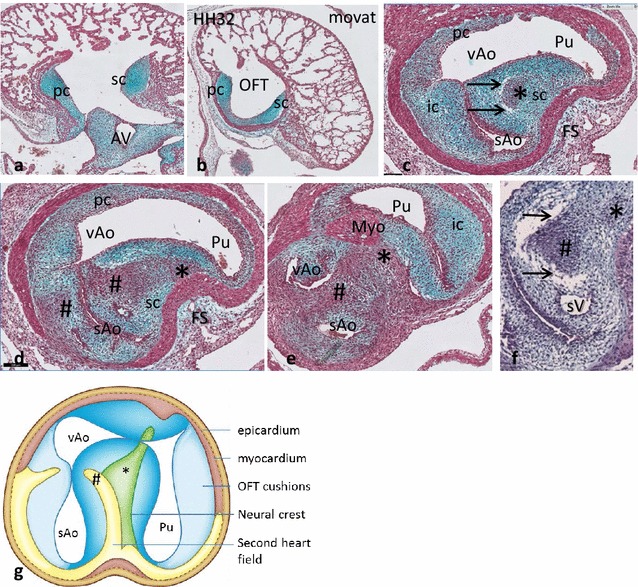
Outflow tract septation in *Crocodylus.* Subsequent levels from the proximal (**a**–**e**) to the distal OFT. **a** The septal and parietal cushion are at the same level as the AV cushions (AV). **b** The OFT is individualized and the parietal and septal cushion form a semicircle. **c** In the distal OFT the sc presents with NCC condensed mesenchyme (*) adjacent to the first vacuoles of the foramen of Panizza (*arrows*). Here the sc is apposed to the intercalated cushion. **d** At this level fusion (#) has occurred between the sc, flanking the folding septum (FS) and the ic separating the systemic from the visceral aorta. **e** Separation of Pu and Ao is completed, here the ventral myocardial protrusion (Myo) meets the NCC-derived condensed mesenchyme and the two aortae are separated by the flow divider (#). *Bar* = 100 µm. **f** Slightly older embryo shows the elaborate vacuolar spaces (*arrows*) proximal to the SHF separation (#) of both aortae, there is not yet a connection with the sinus of Valsalva (sV). **g** Cartoon depicts the division of the 4 distal OFT cushions over the 3 main arterial trunks. Note that only the sAO has two cushion derivatives, while the vAo and the Pu have two large cushions plus one small cushion derivative each. The condensed mesenchyme (*) and aortic flow divider (#) are indicated. For clarity all endocardial cushions are shown in one level. Abbreviations as in Fig. 3

In **HH32** in the proximal OFT, both the septal and parietal cushions contain precartilaginous mesenchyme (Fig. 8a, b). The first stages of formation of the foramen of Panizza (FOP) are seen in the form of a labyrinth of extracellular spaces appearing inside the septal cushion adjacent to the sinuses of Valsalva. These spaces are located in the upstream margin of the aortic flow divider between the systemic and visceral aorta (Fig. 8c, and a slightly older embryo in Fig. 8f indicated by arrows) tunnelling from right to left. The FOP does not yet connect the facing sinuses of Valsalva from the right systemic and left visceral aorta (Fig. 8d).

The pulmonary trunk is embraced by a ventral and by a dorsal myocardial protrusion into the fused septal/parietal cushion that flanks the continuation of the folding septum (Fig. 8d, e). The two inward protrusions of myocardium almost meet in the centre, but are separated by condensed mesenchyme, presumably NCC-derived (compare Fig. 8d, e, *). The aortic flow divider also extends from the septal cushion but in this case towards the right intercalated cushion (as in *Pogona* and *Pelodiscus*), thereby separating the two aortae (#). Coronary ostia above the septal and intercalated cushion of the right systemic aorta are evident from this stage onwards. The total number of ostia observed in a specimen is 4–5.

### Formation of the semilunar valves

The three separated arterial trunks show a different arrangement of endocardial cushions (Fig. 8g). The large septal cushion delivers valve elements to each of the three arterial trunks, similar to what was observed in *Pelodiscu*s. In summary, the right systemic aorta contains two large cushions, whereas the left visceral aorta and the pulmonary trunk each contain two large cushions and a small one. Note that for easy understanding relevant 3D levels are combined in one 2D cartoon in Fig. 8g.

**Fig. 9:**
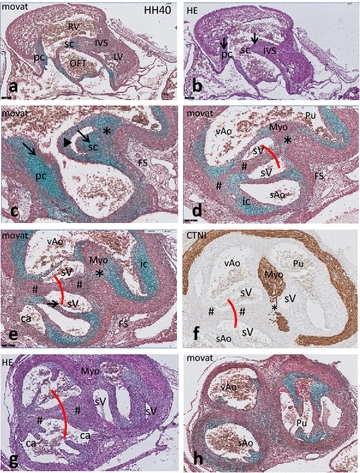
Outflow tract septation in *Crocodylus* HH40. Consecutive sections from proximal to distal (**a**–**h**) stained as indicated. **a** The interventricular septum is indicated but *left* and *right* ventricles are not completely separated, yet. *Bar* = 200 µm. **b**, **c** Sc and pc in the proximal OFT, showing signs of chondrification (*arrows*). **c** Foramen of Panizza (*arrowhead*) is present in sc, flanking the folding septum and adjacent to the NCC condensed mesenchyme (*). **d** Fusion of sc and the ic results in separation of both aortae by the aortic flow divider (#). The facing sinus of Valsalva (*red curve*) are connected with each other through the foramen of Panizza in **c**. *Bar* = 100 µm. **d**, **e** The folding septum and the condensed mesenchyme extend into the lumen between Pu and vAo. Note that some NCC condensed mesenchyme still separates the protrusion of the ventral myocardium (Myo) from the dorsal folding septum [19]. Two adjacent coronary ostia are indicated (ca). **f** CTNI staining demonstrates that the central mass (brown) of **d**–**e** is indeed myocardium, compare with Fig. 8e. **g** The central mass of myocardium (Myo) is continuous with the ventral myocardium. The connection of both aortic valve leaflets is to the left-sided free wall (#). Two further coronary ostia (ca) are indicated. **h** The pulmonary semilunar valve leaflets are located more distal than the aortic leaflets. *ca* coronary ostium, *ic* intercalated cushion, *FS* folding septum, *IVS* interventricular septum, *LV* left ventricle, *Myo* myocardium, *pc* parietal cushion, *Pu* pulmonary trunk, *RV* right ventricle, *sAo* right systemic aorta, *sc* septal cushion, *vAo* left visceral aorta, * NCC condensed mesenchyme, # interaortic flow divider, *arrowhead* foramen of Panizza, *red curved line*: facing sinus of Valsalva in sc, connected by FOP

Even in the oldest stage studied **(HH40)** the interventricular septum is not yet completed, and so a wide communication is still present between left and right ventricular OFTs. The proximal part of the septal cushion (Fig. 9a) is situated between the left and right OFT and shows chondrification. The parietal cushion is also chondrified but to a lesser extent (Fig. 9b, c). The cartilage in the septal cushion extends towards the foramen of Panizza, which in this stage is clearly visible as a tunnel, connecting the visceral and systemic aortic lumina by connecting the facing sinuses of Valsalva of both aortae (Fig. 9d–g). Several coronary arterial entry sites are visible in the facing (Fig. 9g) and non-facing (Fig. 9e, g) sinuses of Valsalva of the systemic aorta.

Septation of the aortae and the pulmonary channels is by involvement of NCC-derived condensed mesenchyme connecting the ventral with the dorsal myocardium (Fig. 9f, g). Condensed mesenchyme is present near the distal tip of the cartilage in the septal cushion bordering the lumen of the foramen of Panizza, flanking the folding septum (Fig. 9c–e). Slightly further downstream the vessel walls are completely separated (Fig. 9g, h) harbouring the valve leaflets. Essentially, the systemic aortic valve is bicuspid (Fig. 9e, g) as is the case with the pulmonary valve (Fig. 9g, h, see also Fig. 8g).

## Discussion

One important consideration in our study is the assumption that our cross-species comparisons are based on comparable stages. It is always difficult to compare stages in different taxa because of confounding factors such as heterochrony [46]. Nonetheless, we can at least use homologous characters such as staining profiles, tissue architecture, endocardial cushion formation and fusion, mesenchymal condensation and cartilage formation [49] to compare developmental sequences in the different species studied. And to assist this task, we used chicken stages as a reference.

### Comparative cardiac septation in reptilian evolution

Cardiac septation within the reptilians and birds (sauropsids) differs significantly among taxa. Lizards and snakes (squamates) and turtles (Testudines) show no ventricular and OFT septation [19], whereas crocodiles and birds (archosaurs) have a biventricular heart with concomitant myocardial OFT separation. We will argue that the squamate heart shows the primitive condition for extant sauropsids, while the archosaur heart is highly derived. The turtle heart more closely resembles the squamate heart [51]. This is perhaps surprising in view of the hypothesis that turtles are a sister group to the archosaurs, the two together constituting the Archelosauria [52]. However, it is possible that the turtles retain the primitive condition because they are, like the squamates, exothermic, and have therefore never evolved the specializations of the heart (including complete ventricular septation) seen in the endothermic mammals and birds, and in the ectothermic crocodilians where the fully septated ventricle may reflect an ancestral endothermic condition for the archosaurs [53].

The birds are unique among the reptiles, and even the amniotes, because the left PAA4 disappears between HH28 and 32, accompanied by apoptosis. The right PAA4 supplies the entire systemic circulation. Even crocodiles, the closest living relatives of birds retain the left PAA4 as a visceral aorta that mostly serves the digestive system. In all species studied here, the right PAA4 is a branch of the systemic aortic trunk from which the carotid trunk branching in the left and right PAA3 and subsequently the subclavian arteries arise. In mammals, the subclavian arteries are branches of the PAA4.

### Endocardial cushions and septation

In this study, the outflow tract is considered to be the myocardial tube with its enclosed endocardial cushions, while the mesenchymal aortic sac is considered to give rise to the arterial trunks. Septation encompasses both the OFT and the aortic sac. Septation in archosaurs involves the formation of a multicomponent complex; homologs of the constitutive elements of this complex can be found, unfused, in turtles and in squamates. Proximally, in early stages of development in *Pogona, Pelodiscus* and *Crocodylus* there is a septal cushion and in addition a layer of cellularized endocardium instead of a parietal cushion. The absence of a parietal cushion in *Pogona* and its relatively late appearance in *Pelodiscus* may be related to the absence of ventricular septation in these species. In later stages (*Pelodiscus, Crocodylus*), both a septal and parietal cushion are present.

Distally, the OFT of all species contains four intramyocardial endocardial cushions of which the dorsally located septal cushion is the largest. The septal cushion continues through the length of the OFT in all species, providing a hemodynamic separation for the pulmonary and aortic channels. The proximo-distal continuation of the other cushions is variable as there are maximal 2 proximal cushions but 4 distal cushions (see Ref. [17] for divergent descriptions). The proximal part of the septal cushion contains the neural crest-derived condensed mesenchyme in chicken. The typical whorl configuration is least evident in *Pogona,* the species that lacks the continuous parietal cushion, and shows no OFT myocardialization at all. Arterial separation remains mesenchymal in *Pogona.* Distal cardiac outflow tract septation shows some myocardialization in *Pelodiscus,* but is only fully myocardialized in *Crocodylus* and *Gallus*. Completion of interventricular septation shows heterochrony when the chicken and crocodile are compared. The oldest crocodile embryo studied (comparable to chicken HH40) still exhibits an interventricular communication that in chicken is closed much earlier, between HH32 and 35.

### Endocardial cushions and semilunar valve formation

The four distal endocardial cushions participate unequally in the formation of the ‘semilunar’ valve leaflets. The large dorsally located septal cushion separates in three parts, one for each arterial trunk. Because of the asymmetric fusion of the septal with the aortic intercalated cushion, the major segment of the latter becomes attributed to the right systemic aorta, whereas a smaller segment is allotted to the left visceral aorta. Again, because of a second asymmetric fusion with the parietal cushion, the major segment of the parietal cushion can be found in the lumen of the visceral aorta, with a smaller segment allotted to the pulmonary trunk, where also the pulmonary intercalated cushion can be found. It is evident that a bicuspid valve is the outcome in all three arterial trunks. The smaller cushion segments probably do not participate in leaflet formation.

In birds and mammals, no asymmetric cushion fusion is observed, and both the dorsal and parietal cushion deliver comparable amounts of cushion tissue to aortic and pulmonary trunks, and together with the intercalated cushion tissue, form arterial valves with three leaflets. In humans, a bicuspid aortic valve is the most common congenital cardiac malformation. The mechanism is poorly understood, but the end result is usually described as an abnormal fusion of cushions/leaflets [54] but may also result from absence of one of the participating endocardial cushions.

### Neural crest, second heart field and septation

It is fortunate that NCC and SHF derivatives could be traced in most reptile species immunohistochemically, at least in early stages, using a combination of TFAP2α for NCC [4], and Isl1 [55] and WT1 staining [56] for the non-NCC. Non-NCC relevant for this area is the SHF cells [2, 6]. We use WT1 as marker for a subpopulation of the splanchnic mesoderm that forms such tissues as the mesothelial lining of the body wall [56], which in turn gives rise to the epicardium and pericardium. The arterial epicardium [14, 15, 57] covers the arterial roots as described here. Whether the WT1 positive mesenchyme in the arterial pole derives from the epicardial epithelium or vice versa is uncertain as WT1 is able to activate both epithelium–mesenchyme transition and mesenchyme–epithelium transition [47]. In organogenesis of the metanephric mesenchyme [48], WT1 regulates gene networks involving Wnt/beta catenin, hedgehog, LRP2, retinoic acid, FGF8/10 and BMP4 signalling, among others. Several of these genes are expressed in the pharyngeal mesoderm, making WT1 a useful marker for SHF cells at the arterial pole.

Chicken and quail-chicken chimeras [6, 10, 16, 58, 59] have provided solid evidence of the NCC and SHF distribution, while genetic markers provided similar evidence in mice [2, 4, 5, 18]. However, in older stages of development the distinction between cell populations is lost, probably due to downregulation of gene expression.

Separation of the two aortic channels is similar in all reptile species investigated here. The involvement of the arterial wall (SHF in the proximal vessel wall and NCC more distally according to chicken and mouse data) is evident in *Pogona, Pelodiscus* and *Crocodylus.* In the crocodile, the intracardiac aorto-pulmonary level is most complex, as it includes the foramen of Panizza, abutted by cartilage [60], channelling through the aortic half of the distal septal cushion and joining the lumina of the visceral and the systemic aorta, but not the pulmonary trunk. Either the NCC or the growing myocardial barrier here is probably preventing the foramen of Panizza from developing in the pulmonary third of the distal septal cushion. The facing semilunar valve leaflets of both aortae covering the outlets of the foramen may serve secondarily as a valve flap, stopping interaortic flow during systole both to prevent high pulmonary pressure and to facilitate a shunting flow during diving in adults [61]. It is reported that in adult crocodiles only the medial leaflet of the right aortic valve covers the foramen during systole, strengthening the idea that blood flow to the brains is favoured even under prolonged diving conditions [61].

In *Gallus*, the situation is different as between HH28 and 32 the left visceral aorta disappears accompanied by apoptosis (see further relevance below), thereby escaping from the septation complex. The consequence is a merging of the interaortic SHF elements (PAA4 flow divider) with the aorto-pulmonary NCC elements (aorto-pulmonary septum) into the definitive aorto-pulmonary septal complex, containing both cell derivatives [10, 59, 62]. It is attractive to search for homologies with mammalian development. We have to realize that in mammals both PAA4 persist, the left one turning into the aortic arch, and the right one that will be incorporated into the right subclavian artery. Mouse OFT development shows that here the subdivision in a right and left PAA4 occurs more downstream than in the reptiles including birds. However, in mammals the interpulmonary flow divider between left and right PAA6 is adjacent to the aorto-pulmonary separation [2] together constructing the aorto-pulmonary septal complex. Aorto-pulmonary separation in mammals and reptiles occurs similarly at the myocardial–arterial junction, but the right PAA6 disappears in mammals. As a consequence, in mammals the aorto-pulmonary septal complex contains both NCC- and SHF-derived elements related to PAA6 [18, 62], whereas in birds the aorto-pulmonary septal complex contains NCC and SHF elements related to PAA4. In the other reptiles, the left aorta is caught between the aortic flow divider and the aorto-pulmonary septum keeping the constituent cell populations mostly separated. Abnormalities in either SHF or NCC may cause a shift in one of the constituents resulting in congenital malformations of the outflow tract and aorto-pulmonary septation [2, 3, 63].

### OFT and apoptosis

Apoptosis in the myocardium and in the mesenchymal AP septal complex including NCC [24, 64–69] has been attributed diverse functions. These include shortening of the OFT, ingrowth of the coronary arteries, separation of the pulmonary and aortic channels, remodelling of the pharyngeal arch arteries (left PAA4 in birds, right PAA6 in mammals), activation of growth factors, and myocardialization of the intracardiac OFT. All these phenomena occur in the same time window, at the same location but in different cell populations. Myocardial apoptosis may be instrumental in, e.g. shortening of the OFT [68] and coronary ingrowth [69], whereas mesenchymal apoptosis may add to aorto-pulmonary separation and myocardialization of the septum [65, 66] or final migration of NCC [67].

In a previous study, we showed a high incidence of apoptosis in embryos concomitant with myocardialization and with the unique disappearance of the left PAA4 and right PAA6 [24]. In *Pogona* lacking both remodelling of the PAA4 and myocardialization, apoptosis was inconspicuous, at a low level in *Pelodiscus*, but more frequent in *Crocodylus* and also in mammals, all with persisting PAAs. It is attractive to bestow signalling by apoptotic NCC a major function in myocardialization of the outflow tract [16, 64].

**Fig. 10:**
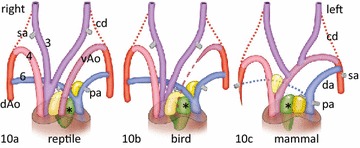
Cartoon depicting the results of aorto-pulmonary septation and PAA remodelling in crocodilians, birds and mammals. **a** In reptiles, represented here by the crocodile, the visceral (*left* PAA4) and systemic aorta (*right* PAA4) are separated by the aorto-aortic flow divider (*light yellow*), while the pulmonary trunk (PAA6) is separated from both aortae by the AP septum (*green*, *). The pulmonary flow divider (*dark yellow*) is not involved in aorto-pulmonary separation. **b** In birds, the left PAA4 disappears and the aortic flow divider merges with the AP septum. **c** In mammals, both PAA4 persist, but the right PAA6 disappears and the pulmonary flow divider merges with the AP septum. In birds, the aorto-pulmonary septal complex results from the merging of the neural crest-derived AP septum with the aortic flow divider and in mammals from the AP septum with the pulmonary flow divider. *AP septum, 3, 4, 6 PAAs by number; *cd* carotid duct, *dAo* dorsal Aorta, *pa* pulmonary artery, *sa* subclavian artery, *vAo* visceral aorta; *Left* left side; *Right* right side

It is evident that the aorto-pulmonary septal complex in the species investigated is different, and this is related to the disappearance of specific pharyngeal arch arteries (Fig. 10). Lizard, turtle and crocodile show no major remodelling of the three arterial trunks and do not combine the AP septum and the flow dividers (Fig. 10a). In birds, the left PAA4 disappears and the aortic flow divider merges with the AP septum to form the avian aorto-pulmonary septal complex (Fig. 10b). In mammals on the other hand the right PAA6 disappears and here the pulmonary flow divider merges with the AP septum to form the mammalian AP septal complex (Fig. 10c).

### Carotid artery differentiation in *Pelodiscus*

The carotid trunk and carotid arch arteries (PAA3) in *Pelodiscus* differ from the other PAAs and arterial trunks with respect to histology. Furthermore, we confirmed the presence of TFAP2α-positive NCC in the cells of the tunica media of the PAA3. In addition, in the crocodile outer cells of the tunica media also expressed TFAP2α, albeit more diffusely. In *Pelodiscus* histology showed a spongious mesenchyme of the tunica media, containing abundant extracellular matrix glycoproteins which is probably less compressible because of the high water content of this matrix. A short survey of available slides [50] of the turtle *E. orbicularis* proved that here this carotid specialization is absent in showing no differences between the various PAA3, 4 or 6. *Pelodiscus* and other soft-shell turtles dive for prolonged times (for instance during hibernation), using buccopharyngeal respiration. Specifically, the buccopharyngeal membrane contains many highly vascularized villi [70]. Increased pharyngeal movements assist in increased O_2_/CO_2_ exchange as well as in urea excretion [71, 72]. These functions probably need some kind of regulation of the blood supply. Functionally, carotid vascular relaxation in these turtles might help to provide the buccopharyngeal area with extra blood, while constriction will turn the haemodynamics to base level. Further studies are needed to examine these issues. The vascular architecture in crocodiles showed no overt differences between PAAs, but crocodiles use the foramen of Panizza as a central shunt between the visceral and systemic aortae, serving another function by providing the brain with additional blood during diving exercise.

### Coronary arterial ostium development in *Crocodylus*

Development of the coronary vascular system depends on many interactions including the sinus venosus-derived endothelium and the epicardium-derived smooth muscle cells [73, 74]. The formation of the stems of the coronary arteries by the ingrowth into the aorta has been described in birds and mammals [75, 76] and is modulated by contact with the aortic endothelium and regulated by differential Tbx1 expression responsible for differences in left/right ingrowth [77]. Usually, in mammals the left and right coronary ostia can be found in the sinus of Valsalva of the left and right leaflet of the aortic semilunar valve, while the aortic intercalated leaflet and the pulmonary trunk are in most cases devoid of a coronary ostium. In reptiles, the coronary arterial circulation differs among taxa [78]. Crocodiles have two aortas, each with bicuspid valve leaflets, and both leaflets of the systemic aorta but not the visceral aorta nor the pulmonary trunk harbour coronary ostia in their sinuses. Future research on such factors as Tbx1 expression patterns in crocodiles could elucidate the mechanisms of coronary ingrowth in a naturally occurring bicuspid aortic valve and shed light on the possible homology of the different endocardial cushions and the leaflets derived from them.

## Conclusions

Outflow tract septation in Amniotes requires the coordinated differentiation of myocardium, endocardium, neural crest and second heart field-derived cells. This takes place in conjunction with the upstream septation of the ventricle and the downstream remodelling of the pharyngeal arch arteries. In lizards and turtles, septation of the ventricle is incomplete, while crocodiles, birds and mammals present with a completely septated left and right ventricle. Early embryos of reptiles including birds present a systemic aorta, emerging from the left-sided (part of the) ventricle providing the main parts of the body. Furthermore, they have a pulmonary trunk providing the lungs and a visceral aorta, mainly for the digestive system. Both emerge from the right-sided (part of the) ventricle. In bird embryos, the left-sided visceral aorta disappears later by apoptosis. In mammals, only the systemic aortic and pulmonary trunks emerge from their respective ventricles, but here the right-sided pulmonary 6th pharyngeal arch artery will disappear early in development. The cells contributing to the septation complexes in the reptiles studied consist of the neural crest-derived aorto-pulmonary septum between aortic and pulmonary trunks. In birds, the second heart field-derived aortic flow divider joins the aorto-pulmonary septum (avian aorto-pulmonary septal complex) as the visceral aorta disappears, while in mammals the pulmonary flow divider will merge with the aorto-pulmonary septum (mammalian aorto-pulmonary septal complex) as the right 6th pharyngeal arch artery will disappear.

In crocodiles, the two persisting aortae have a connection, the foramen of Panizza, allowing shunting of blood, while in the lizard and turtle the shunting occurs at the level of the non-septated ventricle. Although turtles, crocodiles and birds are grouped in the recently formed clade of the Archelosauria, cardiac development of the turtle resembles more closely that of the lizard.
